# Whole‐genome analysis reveals the hybrid formation of Chinese indigenous DHB pig following human migration

**DOI:** 10.1111/eva.13366

**Published:** 2022-03-16

**Authors:** Yuzhan Wang, Chunyuan Zhang, Yebo Peng, Xinyu Cai, Xiaoxiang Hu, Mirte Bosse, Yiqiang Zhao

**Affiliations:** ^1^ State Key Laboratory of Agrobiotechnology College of Biological Sciences China Agricultural University Beijing China; ^2^ Animal Breeding and Genomics Centre Wageningen University Wageningen The Netherlands

**Keywords:** body weight, DaHuaBai pig, human migration, hybridization, *PCM1*, two‐step introgression

## Abstract

Hybridization is widespread in nature and is a valuable tool in domestic breeding. The DHB (DaHuaBai) pig in South China is the product of such a breeding strategy, resulting in increased body weight compared with other pigs in the surrounding area. We analyzed genomic data from 20 Chinese pig breeds and investigated the genomic architecture after breed formation of DHB. The breed showed inconsistency in genotype and body weight phenotype, in line with selection after hybridization. By quantifying introgression with a haplotype‐based approach, we proposed a two‐step introgression from large‐sized pigs into small‐sized pigs to produce DHB, consistent with the human migration events in Chinese history. Combining with gene prioritization and allele frequency analysis, we identify candidate genes that showed selection after introgression and that may affect body weight, such as *IGF1R*, *SRC*, and *PCM1*. Our research provides an example of a hybrid formation of domestic breeds along with human migration patterns.

## 1 INTRODUCTION

Genetic introgression may result in the transmission of beneficial alleles. A growing body of evidence suggests a substantial role of introgression in improving domesticated breeds (Ackermann et al., 2019; Burgarella et al., 2019; Eizirik & Trindade, 2021). The introgression from yak enabled Tibetan taurine cattle to adapt to the extreme environment of the Qinghai–Tibet Plateau (Chen, Cai, et al., 2018; Qiu et al., 2012). Adaptive introgression can also occur between wild animals and domesticated breeds (Scheu et al., 2015; vonHoldt et al., 2010). Cao et al. (2021) found that introgression of wild sheep into domesticated sheep contributed to improving the ability of domestic sheep to resist pneumonia. Although many adaptive introgressions are related to environmental challenges, human‐mediated genetic introgression among pig populations is well documented in commercial breeding. Chinese indigenous pigs, such as Meishan, have contributed to European pig breeds since the nineteenth century (Rothschild et al., 1996; Zhao et al., 2018). Bosse et al. (2015) found that beneficial haplotype allele within the *AHR* locus from Asian pigs was favored via artificial selection after introgression and has played an important role in increasing sow fertility in European pigs.

China is one of the pig domestication centers (Larson et al., 2005). Recent studies suggest that there are possibly multiple domestication locations in China, and it is likely that there has been genetic exchange between different breeds since initial domestication and breed formation (Cai et al., 2019; Jin et al., 2012; Larson et al., 2010; Xiang et al., 2017; Yang et al., 2011). DHB (DaHuaBai) is a local breed in Guangdong province with a distinct high body weight compared with other breeds in South China. The exact origin of the DHB breed has not yet been molecularly resolved. According to the book *Animal Genetics Resources in China*: *Pigs* (Wang et al., 2011), the establishment of the DHB breed may have involved human migration events from the north to the south about 1000 years ago and the immigrants (AD 907–967) to the HeQu river area of Lianzhou, Guangdong province, from Hunan Province, and the immigrants (AD 1127–1279) to the northern Guangdong Province from Jiangxi Province. DHB pig are suspected to be crosses of pigs from the north brought by the immigrants to hybridize with the local pigs in the south. This hypothesis is consistent with that the body weight of DHB is significantly higher than that of other pigs in Southern China, but is comparable with that of pigs from Northern China.

Due to the distinct body weight phenotype of the DHB breed and relevant historical documentation of human migration, it is hypothesized that the genome of DHB not only retains the signals of genetic introgression from pigs in the north to pigs in the south, but also it preferentially retains gene variants transmitted from the north that are involved in increased body weight. From this perspective, DHB constitutes an excellent model for studying human‐mediated migration and introgression of Chinese indigenous pigs. Unfortunately, although there are as many as 100 more pig breeds recorded in the book of Wang et al. (2011), there is currently no systematic research on the origins and histories of Chinese indigenous pigs at the molecular level, including for DHB.

In this study, we used large‐scale genotype and resequencing data to infer the origin and hybrid history of the DHB breed. We reported a possible two‐step introgression to produce DHB along with human migration. Genes and variants that potentially affect phenotypes in DHB were identified via introgression and functional analysis, thus contributing to the large body weight of the breed. Together, the DHB breed demonstrates how genomic patterns reflect the history of human breeding efforts in creating a novel composite breed.

## 2 MATERIALS AND METHODS

### 2.1 SNP filtering and phasing

Chip data used in this study were retrieved from Yang et al. (2017). The data contain genotypes for 61,772 SNPs in 3482 individuals from 146 different pig breeds. We used probeID (https://support.illumina.com/array/array_kits/porcinesnp60_dna_analysis_kit/downloads.html) to convert the original Sscrofa10.2 genome coordinates to coordinates of Sscrofa11.1. We used plink1.9 (Chang et al., 2015) to filter the data (‐‐mind 0.1: filters out all samples with missing call rates exceeding 0.1; ‐‐geno 0.1: filters out all variants with missing call rates exceeding 0.1), and bcftools v1.10 (Danecek et al., 2021) to remove duplicate sites. After filtering, the genotyping rate of chip data was 0.9956. BEAGLE5 was used to phase (Browning & Browning, 2007) and impute (Browning et al., 2018) the entirety of SNP data with default parameters. After phasing and imputing, the complete chip dataset contains 54,323 SNPs and 2,102 individuals, but we only used 53,066 autosomal SNPs and a subset of domestic pigs from China. Semi‐wild breeds, including pigs from Tibetan or Yunnan, as well as others without clear body weight records, were also excluded. Finally, we extracted 20 Chinese pig breeds and SBSB (*Sus barbatus*) from the processed data for further analysis.

### 2.2 Population structure and phylogenetic analysis

The body weight information and illustrations of Chinese local pig breeds were retrieved from the book by Wang et al. (2011). The IBS distance matrix between individuals was calculated using plink1.9 (‐‐genome; ‐‐distance‐matrix). The distance matrix was used to build an unrooted neighbor‐joining tree by FastME (Lefort et al., 2015); iTol (Letunic & Bork, 2019) was used for tree display. We used plink1.9 to prune the chip data with the parameter of ‐‐indep‐pairwise 50 10 0.2. Principal component analysis (PCA) for the 20 Chinese breeds was performed using SMARTPCA (Patterson et al., 2006) based on the LD‐pruned data. Admixture (Alexander et al., 2009) analysis was performed on the LD‐pruned data using the program ADMIXTURE (v1.23), by varying *K* = 2–20. The admixture results were plotted using pophelper R package (Francis, 2017). The relationship between global ancestry estimated from admixture and body weight of pig breeds was assessed by linear regression. Cook's distance was then used to pinpoint the influential data points.

### 2.3 *D*‐statistic test of genetic introgression between DHB and large‐sized breeds

*D*‐statistic analysis was performed using qpDstat (AdmixTools) (Patterson et al., 2012) on DHB, small‐sized breeds, large‐sized breeds, and *Sus barbatus* as an outgroup using the combination *D* (DHB, small‐sized breeds, large‐sized breeds, *Sus barbatus*). A positive *D*‐statistic with a *Z*‐score of >3 indicated gene flow between DHB and large‐sized breeds. All eligible combinations and corresponding *D*‐statistic values were plotted.

### 2.4 Genome‐wide introgression detection based on haplotype similarity

Considering that the choice of block sizes may affect results, we determined the haplotype block size by measuring the linkage disequilibrium (LD) of the DHB population. We found the highest fraction of blocks showing average LD of greater than 0.2 in blocks of five SNPs (Table S2). The genome (53,066 SNPs) was thus divided into 10,619 nonoverlapping blocks with a fixed number of five adjacent SNPs. To detect introgression signals, we adopted the method proposed by Zhang et al. (2019). If extensive introgression occurred from large‐sized breeds into DHB, we would observe genomic loci of DHB exhibiting higher haplotype similarity to large‐sized breeds than to small‐sized breeds. Haplotype similarity was evaluated by cumulative Chi‐squared statistics between DHB versus large‐sized breeds and DHB versus small‐sized breeds:

$$\chi^{2}=\sum_{i=1}^{n}\frac{{\left(A_{i}‐T_{i}\right)}^{2}}{T_{i}}$$

where *A_i_* and *T_i_* are observed, and expected blocks count for cell *i*. Overall, the higher the similarity between the two populations, the lower the Chi‐square values. We then constructed Δχ^2^ as a Chi‐square between DHB and large‐sized breeds minus a Chi‐square between DHB and small‐sized breeds:

$$\Delta x^{2}=\chi_{\mathrm{DHB}\;\mathrm{vs}.\;\;\mathrm{Small}}^{2}‐\chi_{\mathrm{DHB}\;\mathrm{vs}.\;\;\mathrm{Large}}^{2}$$

To assess the significance, we performed permutation tests for each block and reported the blocks with a *p* value of ≤0.001 (Zhang et al., 2019). Each large‐sized breed was tested separately with each of the four small‐sized breeds.

To test whether the blocks of introgression from different large‐sized breeds to DHB were randomly distributed across the genome, we simulated the data by randomly selecting an equal number of introgression blocks identified for each large‐sized breed from the 10,619 blocks and then calculated overlapping blocks for each paired large‐sized breed. The process was repeated 1000 times to construct a null distribution. The significance was estimated by comparing the real number against the 97.5% quantile of the null distribution.

### 2.5 Selection signal analysis of the introgression regions

An in‐house script was used to calculate the *H*12 statistic (Garud & Rosenberg, 2015) for each introgressed block in small‐sized pigs and DHB. The formula of the *H*12 statistic is as follows:

$$H12={\left(p_{1}+p_{2}\right)}^{2}+\sum_{i=3}^{\infty }p_{i}^{2}$$

where $p_{i}$ represents different types of haplotypes, with $\sum_{i=1}^{\infty }p_{i}=1$ and $p_{1}\geq p_{2}\geq p_{3}\geq p_{4}\geq ..>0$

Vcftools (v0.1.13) (Danecek et al., 2011) was used to calculate the genome‐wide *F*_st_ at each SNP between DHB and small‐sized breeds; the top 5% differentiation sites were considered as under selection. The Fisher's exact test was used to determine whether or not there are significantly more blocks under selection in the introgression regions compared with other regions in the DHB genome.

### 2.6 GO enrichment and gene prioritization analysis

The human orthologs of pig genes were retrieved from BioMart (Ensembl) (Howe et al., 2020) for GO enrichment analysis, which was performed using metascape (Zhou et al., 2019) based on the human gene annotation database. We collected a set of 489 ortholog genes associated with human body weight from GWAS studies (https://www.ebi.ac.uk/gwas/efotraits/EFO_0004338) as training genes. Endeavour (Tranchevent et al., 2016) was used to prioritize the introgression genes, given the collected training genes.

### 2.7 Construction of the demography model

We used the approximate Bayesian computation approach (ABCToolbox) (Wegmann et al., 2010) to fit a suitable model that can explain the demographic history of the DHB. Before ABC inference, some SNPs were removed by three criteria: first, SNPs located in CDS or within 100 kb distance to CDS; second, SNPs located in CpG islands; third, SNPs subjected to LD pruning by plink using “‐‐indep‐pairwise 50 10 0.2” (Frantz et al., 2015). Finally, we obtained 8,004 independent and putatively neutral SNPs for ABC inference. We included population statistics of ALL_H (mean heterozygosity for all populations), ALLSD_H (standard deviation of heterozygosity for all populations), MEAN_H (mean heterozygosity for each population), SD_H (standard deviation of heterozygosity for each population), and PAIRWISE_FST (pairwise *F*_st_) in the model. ABCsampler and fastsimcoal26 were used to construct the model and to perform coalescent simulations. The observed and simulated summary statistics were calculated using arlsumstat. For each model, half a million replicates were simulated. ABCestimator was then used to calculate the marginal posterior distribution from 5000 (1%) retained simulations by the general linear model. Observed summary statistics located within the 95% interval of the retained simulated statistics indicate a good model fit. Bayes factor, which is usually used for model selection, was computed by comparing the marginal density estimated from ABCestimator for two models.

### 2.8 Whole‐genome sequencing analysis

Whole‐genome sequencing data of 80 samples from 12 Chinese pig breeds (Table S13) were downloaded from the NCBI SRA database. We sequenced an additional six DHB pig and six SZL pigs (coverage depth: 7X‐26X). For each sample, we used the fastp tool (Chen et al., 2018) with default parameters for quality filtering. We generated gVCF for each sample against the reference genome of Sscrofa11.1 (GenBank accession: GCA_000003025.6) and performed joint calling for all individuals using the GTX‐One platform, which is an FPGA‐based hardware accelerator for GATK best practices workflow (Bu et al., 2020). We finally filtered out SNPs by setting the parameters of QD < 2.0, MQ < 40.0, FS > 60.0, SOR > 3.0, MQRankSum < −12.5, ReadPosRankSum < −8.0, and QUAL < 30 using GATK (4.1.9.0) VariantFiltration (DePristo et al., 2011).

For introgression regions in the DHB genome identified by the chip data, we recorded the genomic coordinates. Vcftools was again used to calculate the genome‐wide *F*_st_ at each SNP between DHB and small‐sized breeds. SNPs that had a missing rate of less than 10% and showed the top 1% of differentiation by *F*_st_ analysis were extracted and annotated by SnpEff (Cingolani et al., 2012).

## 3 RESULTS

### 3.1 Inconsistency of genotype and body weight phenotype of DHB

**FIGURE 1:**
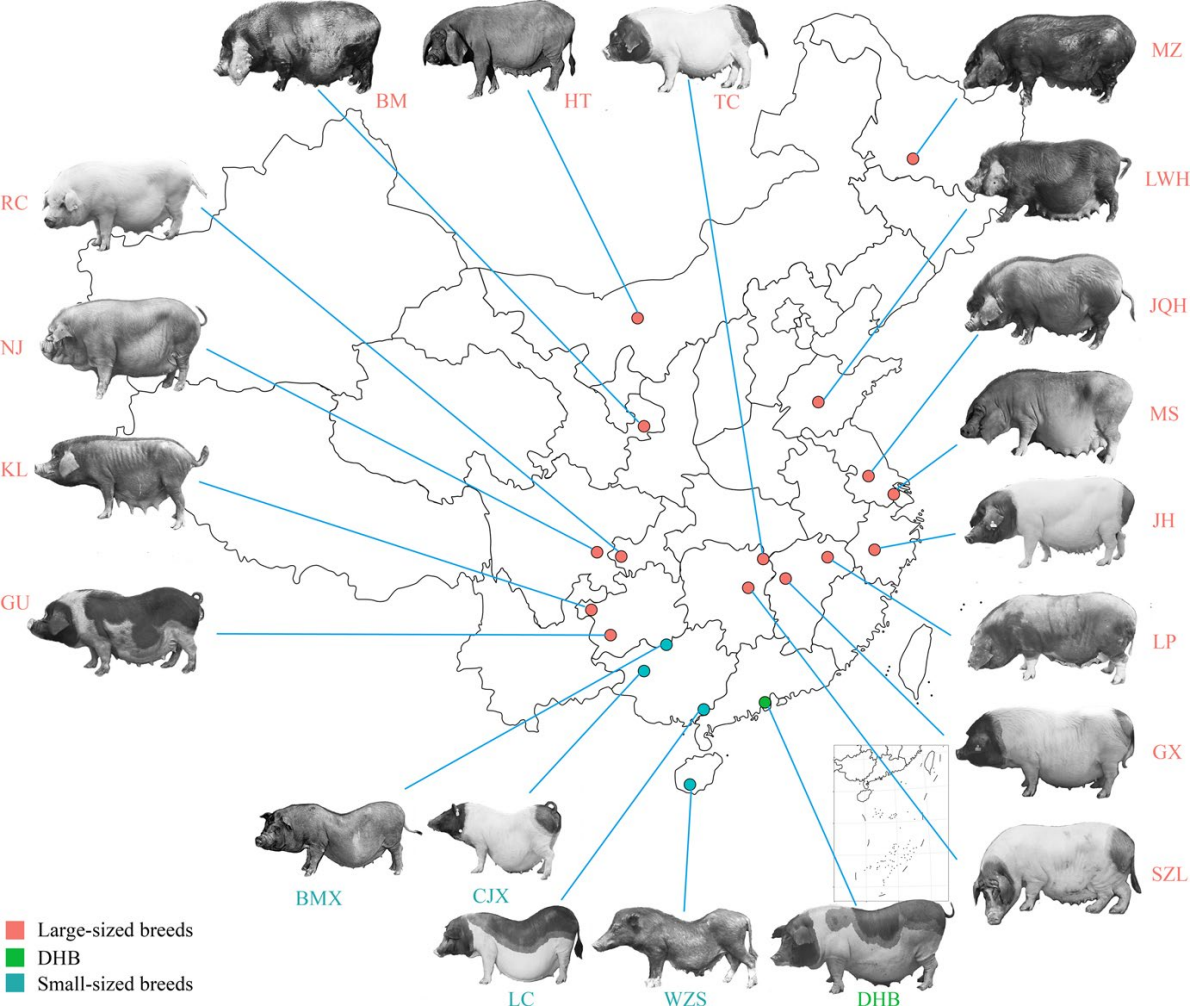
Distribution of Chinese local pig breeds included in this study. Large‐sized (northern) breeds are abbreviated in red, small‐sized (southern) breeds are abbreviated in dark green, and DHB is abbreviated in light green. The geographical locations of pig breeds were retrieved from Yang et al. (2017)

**FIGURE 2:**
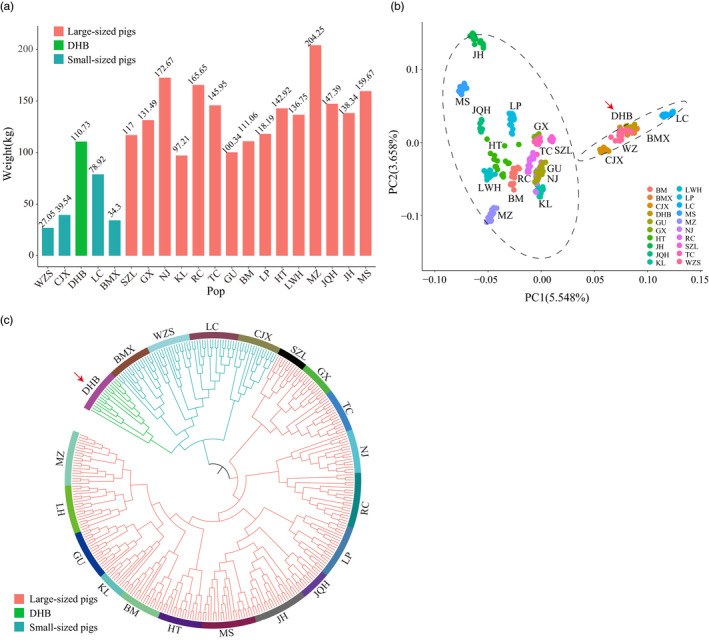
DHB was clustered differently in terms of body weight and genetic composition. (a) Body weight of the 20 Chinese local pig breeds. Body weight is the sow weight of the breed collected from the book of Wang et al. (2011). (b) PCA projection of pig breeds using whole‐genome SNPs. PC1 and PC2 explained 5.55% and 3.65% genetic variance, respectively. 95% confidence ellipses were drawn for the two groups of large‐sized and small‐sized breeds. (c) NJ tree based on IBS distance matrix of all pig individuals

In this study, we included 338 individuals from 15 Chinese large‐sized breeds, four Chinese small‐sized breeds, the DHB (DaHuaBai) breed, and SBSB (*Sus barbatus*) as the outgroup (Figure 1, Table S1). All individuals were genotyped using the Illumina Porcine 60k Bead Chip. In total, 53,066 SNPs that passed our quality control were kept for further analyses (see Materials and Methods). Geographically, LC (LuChuan), BMX (BaMaXiang), CJX (CongJiangXiaoEr), WZS (WuZhiShan), and DHB are from South China. SZL (ShaZiLing), GX (GanXiLiangTouWu), TC (TongCheng), LP (LePing) are from Central China. KL (KeLe), NJ (NeiJiang), RC (RongChang), GU (GuanLing) are from Northwest China. BM (BaMei), HT (HeTao), LWH (LaiWuHei), MZ (MinZhu) are from North China. JQH (JiangQuHai), JH (JinHua) and MS (MeiShan) are from East China (Figure 1). Except for DHB, the four breeds from the South are all small‐sized, with an average body weight of no more than 80 kg (WZS: 27.05 kg, CJX: 39.54 kg, LC: 78.92 kg, and BMX: 34.4 kg). In contrast, pig breeds from the other regions, except for South China, are large‐sized with an average body weight almost above 100 kg (e.g., GX: 131.49 kg, JH: 138.35 kg, and SZL: 117.01 kg) (Figure 2a). The body weight of DHB falls in the large‐sized group, with an average of 110.73kg. Since the large‐sized breeds were geographically located to the north of the small‐sized breeds, in this study, we called the large‐sized breeds northern breeds and the small‐sized breeds southern breeds, unless otherwise specified.

Using the chip SNP data, we conducted PCA (principal component analysis) on the 20 Chinese pig populations (see Materials and Methods; Figure 2b; Figure S1). The PCA clustering result is similar according to the geographical locations. The group on the left contained 15 large‐sized breeds (or pig breeds from North China), while the group on the right contained four small‐sized breeds (or pig breeds from South China) plus DHB. The neighbor‐joining tree based on the IBS distance matrix (Figure 2c) corroborated that, at the genetic level, the small‐sized breeds plus the DHB form a monophyletic cluster. In contrast, the large‐sized breeds were clustered into another branch.

In summary, although DHB belongs to the genetic cluster of small‐sized breeds from South China, their larger body weight, together with the historical documentation of human migration, suggested potential crossbreeding with the northern pigs.

### 3.2 Genomic evidence of hybridization between large‐sized breeds from the North and small‐sized breeds from the South to produce DHB

**FIGURE 3:**
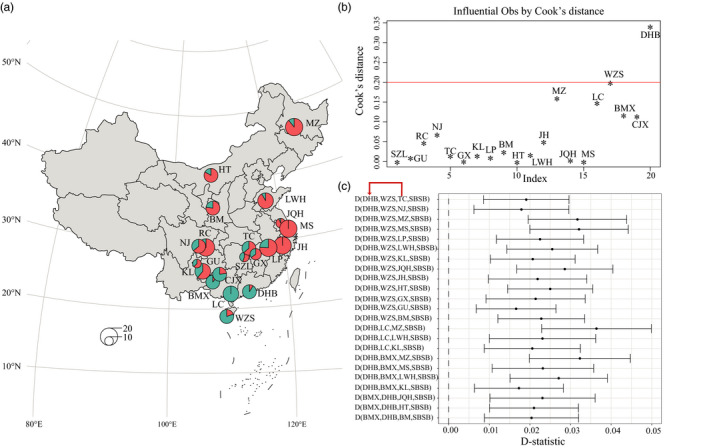
Genetic evidence of hybridization of DHB. (a) Genetic structure of pig breeds for *K* = 2. The average ancestral composition of each breed was averaged for all individuals of the breed. The sample size of the breed determined the size of the pie chart. (b) Cook's distance identified DHB as the most influential outliers in the regression model. The red line indicates the threshold of 4/n, where n is the number of breeds. (c) *D*‐statistic analysis with *D* (DHB, small‐sized breeds, large‐sized breeds, SBSB) (*y*‐axis) for detection introgression from large‐sized breeds into DHB. Four small‐sized breeds and 15 large‐sized breeds formed 60 *D*‐statistic test combinations

Considering the contradictory classification of DHB with respect to body weight and genotype, one possibility is that the genetic makeup of DHB is a mixture derived from large‐sized and small‐sized breeds, with the majority of their genetic composition coming from small‐sized breeds. We conducted admixture analysis for all 20 Chinese breeds using *K* = 2–20 (Figure S2). When *K* = 2, the genetic composition was mostly reflected by geographical location (Figure 3a). As shown in Figure 3a, the genetic composition of LC (in green) was 99% pure; thus, we used LC as a proxy for unadmixed small‐sized breeds, or southern breeds. Similarly, we used MS and JH as proxies for unadmixed large‐sized breeds, or northern breeds, of which the genetic composition of MS and JH (in red) is 99% pure. Small‐sized breeds such as BMX, WZS, and CJX share 89%, 80%, and 77% ancestral composition with LC, respectively. Other large‐sized breeds share ancestral composition with MS and JH from 55% to 93%. When plotting the genetic ancestry composition onto the map, as shown in Figure 3a, it was evident that two distinct ancestral groups existed. Pig breeds with mixed genetic composition were geographically located in‐between.

Although the body weight of DHB approaches that of large‐sized breeds, admixture results showed that the ancestral composition of DHB was highly shared with LC (91%) but less shared with MS and JH (9%). We next regressed the body weight of all breeds against their ancestry ratio shared with LC (admixture analysis *K* = 2). A significant negative correlation was observed, with a Pearson correlation coefficient of −0.748 (95% CI: −0.894 to (−0.457)) (Figure S3). Cook's distance analysis identified the DHB as the most influential outlier affecting the regression model (Cook's distance: 0.3245; Figure 3b), suggesting that the specific genetic material in DHB was highly associated with increased body weight.

To perform a formal test of genetic introgression from large‐sized breeds into DHB, *D*‐statistic was used to detect possible introgression in 60 combinations (15 large‐sized breeds versus 4 small‐sized breeds), using SBSB as an outgroup, and small‐sized breeds (either for WZS, BMX, LC, or CJX) and DHB as sibling groups. We found that 23 out of 60 tests supported introgression to DHB with confidence (Figure 3c).

**FIGURE 4:**
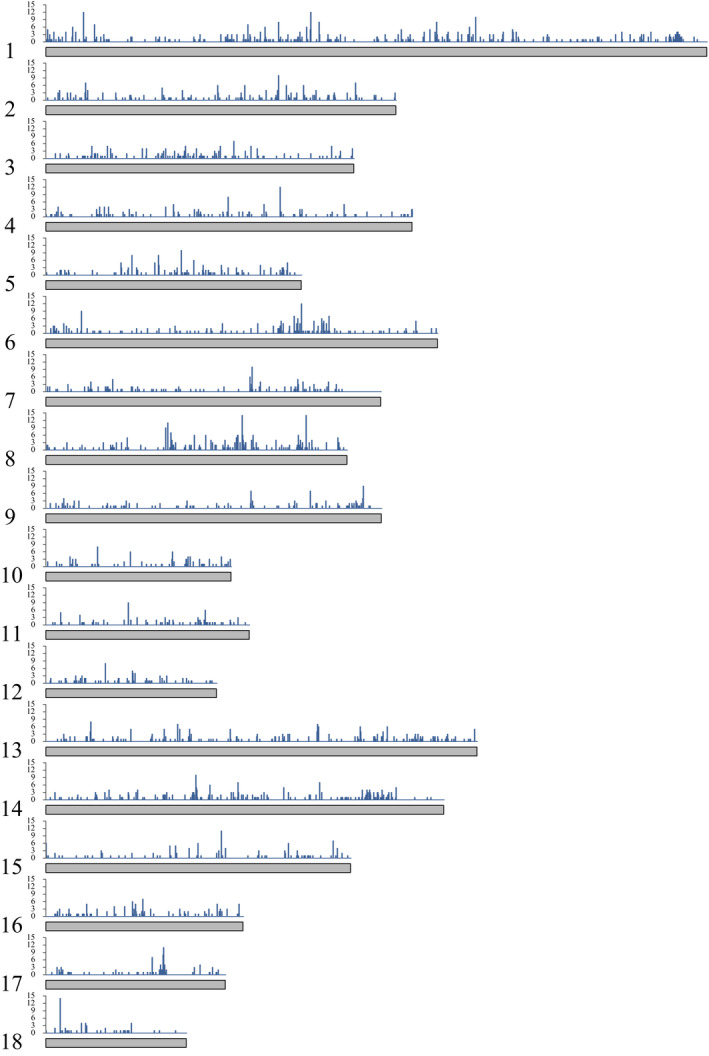
Genome‐wide introgression map of DHB from large‐sized breeds. Only data from chromosomes 1–18 are plotted. The *y*‐axis indicates the number of large‐sized breeds that had introgressed into DHB

To gain detailed insights into the introgression of large‐sized breeds into the DHB, we sought genomic regions in the DHB genome that were genetically closer to large‐sized breeds and more distant to small‐sized breeds, based on a haplotype similarity approach developed by Zhang et al. (2019) (see Materials and Methods), and we constructed a genome‐wide introgression map of DHB (Figure 4; Table S3). Consistent with the *D*‐statistics results, among the introgression regions, GX showed the most extensive introgression into DHB, with summed introgression lengths of 58 MB in total. SZL (48 MB), JH (40 MB), JQH (53 MB), and MS (42 MB) also showed a high degree of introgression to DHB (Figure S4).

### 3.3 Model of two‐step introgression to DHB

The *H*12 statistic is widely used for measuring haplotype diversity. It calculates the sum of the squares of haplotype frequencies, combining the two most common haplotypes into a single frequency (see Materials and Methods). We found higher values of *H*12 in DHB than in most small‐sized breeds in the introgression regions (Table S4). We also calculated genome‐wide *F*_st_ between DHB and small‐sized breeds and found significantly more blocks under selection in the introgression regions, compared with blocks in the nonintrogression regions (Fisher's exact test, *p* < 0.01; Table S5). Together, this evidence indicated that the introgression regions we identified in the DHB genome were under positive selection.

**FIGURE 5:**
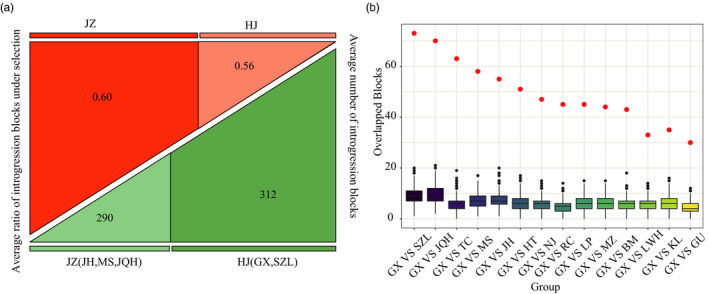
Genetic evidence of two‐step introgression to DHB. (a) Pig breeds with a higher degree of introgression were less constrained in the introgressed region. We divided the top five pig breeds with highest introgression into two groups according to their geographic location, namely JZ (JH, MS, and JQH) and HJ (GX and SZL), and calculated the average number of introgression blocks (in green) and the ratio of the introgression blocks under selection, as detected by top 5% *F*_st_ value (in red) for the two groups. (b) Observed and expected overlapped introgression blocks between GX and other large‐sized breeds. GX served as an intermediate introgression in the two‐step introgression chain. The box diagram represents the random sampling distribution from 1000 simulations; red dots indicate the number of overlapped blocks observed

The introgression blocks shared between paired large‐sized breeds were highly overlapped compared with random expectation (see Materials and Methods; Figure S5). By investigating the number and length of blocks of introgression from different large‐sized breeds, we noticed that the large‐sized breeds geographically closer to DHB displayed gradually more introgression length (Figure S4). This pattern of gradual introgression also excluded the possibility that the regions shared by multiple large‐sized breeds were caused by incomplete lineage sorting due to random chance. However, we found that pig breeds with a higher degree of introgression tended to be less constrained when checking the ratio of introgressed blocks under selection (Figure 5a; Table S6). We hypothesized that this was due to a chain‐manner introgression from the north to the south, with the transmission of beneficial alleles originally from the north because more extensive and noisy introgressions are expected to happen at the end of the introgression chain. As the GX seemed to be the last step in the introgression route to DHB, we also assessed the overlap of introgression regions between GX and other large‐sized breeds that were possibly in the introgression chain (Figure 5b). We observed significantly more overlapped introgressions with GX and these breeds than random expectation (Figure S5, GX, and SZL: 63.97 more blocks, GX and JQH: 60.19 more blocks, GX and MS: 50.93 more blocks, GX and JH: 47.68 more blocks). From a geographical point of view, GX is located in Jiangxi Province and SZL is located in Hunan Province, and both are geographically closer to DHB. JH, JHQ, and MS are located in Jiangsu and Zhejiang province, which are geographically distant from DHB but are next to SZL and GX. Our observation agreed with the "chain introgression" hypothesis, in which pig breeds from Jiangsu or Zhejiang in the north initially introgressed into pig breeds in Hunan or Jiangxi in the middle, followed by a second wave of introgression to the South that produced DHB.

**FIGURE 6:**
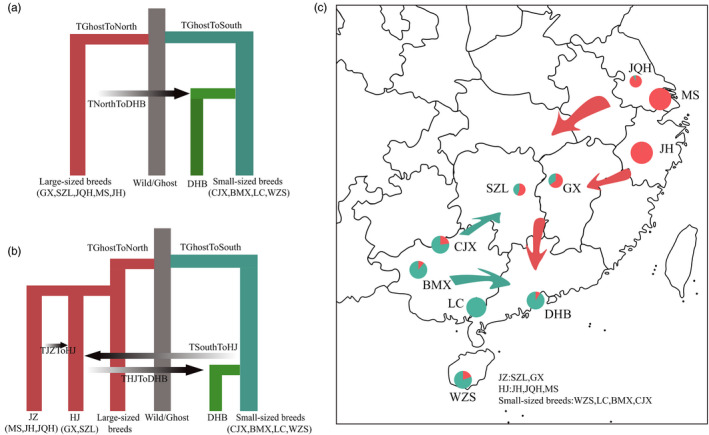
Demographic model of DHB breed formation. (a) Simple demographic model of DHB breed formation. In this model, DHB was derived from small‐sized breeds from the south with introgression of pigs from the north. (b) A two‐step introgression demographic model of DHB breed formation. In this model, GX and SZL served as agents for initial introgression, and a second wave of introgression from GX and SZL migrated to the south and produced DHB. (c) Breeds migration route map. The color and size of the pie chart are the same as in Figure 2a

We next used an approximate Bayesian approach and coalescent simulation to build differentiation models for DHB. With our prior knowledge of ancestral composition and the introgression pattern of DHB, we could design the model more efficiently. In our first model, the Chinese domestic pigs were divided into north and south groups. All four small‐sized breeds were grouped into one group, and the top five introgression large‐sized breeds (GX, SZL, JH, JQH, and MS) were grouped into the other group. The large‐sized breeds from the north and small‐sized breeds from the south were differentiated from a common ancestor. The result of the *D*‐statistic showed that there was genetic introgression between large‐sized breeds and DHB, but the direction was not determined. Therefore, we designed two models with opposite introgression directions. Model 1 (Figure 6a) was designed based on our findings that DHB is genetically close to the small‐sized breeds, while possibly having been introgressed from large‐sized breeds. In Model 2 (Figure S6), the group of breeds was consistent with Model 1 (Figure 6a); the difference was in the direction of introgression, from DHB into large‐sized breeds. The Bayes factor obtained by ABCestimator was 1.9, which indicated that the model shown in Figure 6a could better fit the observations compared with Model 2 (Figure S6). As shown in Figure 6a, DHB differentiated from the pigs from the south, along with the migration of pigs from the north to the south. Model 1 suggested that the establishment of the DHB breed started from 1558.76 years ago (50% highest posterior density intervals (HPD): 1165.06–1828.83) with 33.86% (50% HPD: 18.80–40.33%) genetic composition initially introgressed from the pigs from the north to the pigs in the south (Table S7; Figure S7a).

Since we suggested a chain‐manner introgression as above, and GX and SZL that are geographically closer to the place where the small‐sized breeds live appeared to be half admixed. In our third model (Figure 6b), we incorporated the scenario of two‐step introgression in which GX and SZL served as agents for genetic introgressions. In this model, we estimated that pigs in Zhejiang migrated to Hunan or Jiangxi about 1200 years ago (50%HPD: 866.05–1449.98), and a second wave of introgression of pigs in Hunan or Jiangxi migrated to the south and produced DHB about 800 years ago (50% HPD: 487.42–1044.56) (Figure 6c; Table S8; Figure S7b).

### 3.4 Functional analysis of introgression genes

Due to the evidence from Cook's distance, selection signals along the introgression route, and the increased body weight of DHB, we expected that the introgressed regions in DHB would harbor important genes controlling body weight. Among the 388 blocks that showed evidence of introgressions from more than three large‐sized breeds, 855 genes were retrieved. GO enrichment analysis revealed that the introgression genes were significantly enriched in muscle contraction (GO: 0006936), blood circulation (GO: 0008015), and cardiac muscle contraction (GO: 0060048) (Table S9).

To obtain a thinned set of best candidates, we prioritized the 855 genes using the Endeavour method, given the known genes associated with human body weight as the training set. In total, 503 genes were successfully prioritized (Table S10). Table 1 lists the top 20 genes based on the prioritized *p* value. Among the best candidates, *IGF1R* encodes for an insulin receptor. Previous studies showed that this gene affected body weight in many species, such as mice, Japanese quail, and cattle (Moe et al., 2007; Szewczuk et al., 2013; Todd et al., 2007). Zhao et al. (2018) also found that the *IGF1R* was selected in Meishan pigs, which may be related to the high fertility of this breed. *SRC* is related to food intake in mice (Yang et al., 2019), and *ADCY2* is reported to affect human subcutaneous fat content, both of which are functionally relevant (Daily et al., 2019).

**TABLE 1:** Top prioritized introgressed genes in the DHB genome

Gene	Chromosome	Block start	Block end	Number of introgressed populations
*IGF1R*	1	137008027	137430730	5
*SRC*	17	40326252	40517532	4
*ADCY2*	16	74541662	74616199	3
*MAP3K7*	1	58446382	58544570	3
*NRIP1*	13	179884140	180051342	3
*FOS*	7	98401765	98508334	3
*RBPJ*	8	20011888	20111114	3
*IL6ST*	16	35149902	35219808	5
*CAMK4*	2	115928254	116128462	10
*DCC*	1	102981316	103118451	3
*PCM1*	17	5557099	5831348	3
*PRKG1*	14	98108806	98237722	7
*PDE4D*	16	37970230	38177214	7
*ZFHX4*	4	59481109	59596863	3
*CAMK2A*	2	151206576	151328030	3
*RABGAP1*	1	263806904	264148357	3
*PRKCQ*	10	64441239	64539431	4
*GRIA1*	16	69441441	69459646	3
*ID2*	3	127438077	127529210	3
*EDNRB*	11	49735789	50089826	3

**FIGURE 7:**
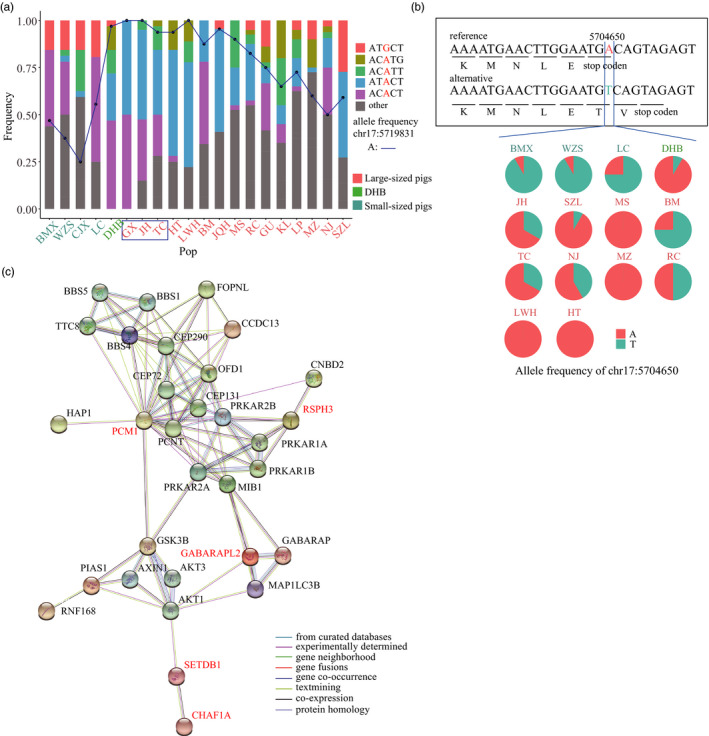
Frequency and functional analysis of introgression gene *PCM1*. (a) Multiple haplotypes co‐existed in introgressed block chr17:555709‐5831348. The frequency of haplotype ATACT was higher in large‐sized pigs and DHB but lower in small‐sized pigs. The blue box indicates that genetic introgression into DHB had been detected in these breeds. Two alleles (AG) were observed at site 5719831bp on chromosome 17, marked in red in the legend. The black dot and the blue line indicate the frequency of the A allele in all breeds. (b) The alternative allele T on chr17:5704650 introduces two extra amino acids in the protein sequence of gene *PCM1* compared to reference allele A. The frequency of this SNP in each population is represented by a pie chart, in which red refers to allele A and blue refers to alternative T. (c) Interaction network centered by *PCM1*; only the top 25 STRING interactants are shown in the figure. Genes in red indicate that they are identified as introgressed genes in this study

Since the chip data only offer a compromised resolution, to investigate candidate mutations in more detail, we collected whole‐genome resequencing data from a public database of 80 Chinese pigs and sequenced six DHB pig and six SZL pigs to scan for functional SNPs in the introgression regions (Materials and Methods). We annotated SNPs in the introgression region with high *F_st_* signals between DHB and small‐sized breeds using SnpEff and identified four SNPs with high‐impact effect (Table 2; Table S11) and 161 SNPs with moderate‐impact effect (Table S11). The *PCM1* gene, which was reported to associate with early‐onset obesity in children, caught our attention (Pettersson et al., 2017). In the chip data, the dominant haplotype (ATACT, chr17:5557099–5831348) in *PCM1* was at a high frequency in DHB and large‐sized breeds, but at a low frequency in small‐sized breeds (Figure 7a). Within the haplotype, the frequency of the allele A at site chr17:5719831 is close to fixed in DHB and most large‐sized pigs, while the allele A frequency is relatively low in small‐sized pigs (Figure 7a). Of the four SNPs that we identified as high‐impact using resequencing data, the reference allele A in position chr17: 5704650, which was at a higher frequency in most large‐sized pigs but at a lower frequency in small‐sized pigs (Figure 7b), produces an early stop codon, causing the *PCM1* protein to be truncated compared with alternative allele T (Materials and Methods). We used the STRING (https://www.string‐db.org/) to extract proteins that are functionally associated with *PCM1* (Figure 7c). Of the 25 proteins in the protein–protein interaction (PPI) network using a *p* value threshold of 10^−16^ (PPI enrichment), *RSPH3*, *GABARAPL2*, *SETDB1*, and *CHAF1A* were also identified as introgressed genes by our haplotype similarity approach (Table S3). Among the genes, *GABARAPL2* is associated with total and high‐density lipoprotein–cholesterol (Capel et al., 2008), and *SETDB1* is an antiadipogenic factor playing a role in obesity (Okamura et al., 2010). These genes, together with other genes with high‐impact variants, are worth further experimental validation.

**TABLE 2:** SNP sites with high impact

Chromosome	Gene	Position	Ref/Alt	Effect
chr2	*LOC100525562*	63348710	T/G	stop_gained
chr3	*IMMT*	58600395	A/G	splice_acceptor_variant&intron_variant
chr13	*MRAP*	195965524	A/G	splice_acceptor_variant&intron_variant
chr17	*PCM1*	5704650	A/T	stop_lost

## 4 DISCUSSION

Most studies on genetic introgression focus on introgression between highly divergent lineages, such as interspecies hybridization or introgression of wild counterparts into domesticated species. Due to incomplete population differentiation, it is more difficult to quantify introgression between evolutionarily closely related breeds within species. By utilizing the haplotype similarity approach, we investigated hybridization between domesticated pig breeds on a relatively short timescale. For the first time, at the molecular level, we studied the origin and history of the DHB breed that seem to be the product of hybridization of large‐sized and small‐sized breeds. Based on the quantification of introgression of multiple breeds, we proposed a two‐step introgression from the north to the south, which is in line with historical records as well as our demographic modeling.

In our demographic model, we inferred that the pigs from Jiangsu and Zhejiang provinces migrated to Hunan and Jiangxi provinces about 1200 years ago (50% HPD: 866.05–1449.98) (AD 552–1135), and a second wave of migration happened about 800 years ago (50% HPD: 487.42–1044.56) (AD 957–1513) from Hunan and Jiangxi to South China (Guangdong and Guangxi province), finally producing the DHB breed. During the second half of the Tang dynasty (AD 755–907), rebellion and uprisings erupted all over the country. The country later broke down into warring states, and short‐lived military regimes alternated frequently, referred to as the "Five Dynasties and Ten Kingdoms" (AD 907–967). During this period, the southern part of China was more stable than the northern area; the economic center of gravity shifted southward as well as the northern population. The years AD 960–1279 corresponded to the Song Dynasty in Chinese history. The Mongols conquered the Song Dynasty at the end of the thirteenth century. A large number of refugees migrated from the north to the south with their production materials, such as local domestic pigs. The inferred timing from ABC reconstruction is highly accordant with the migration history in China and also verifies the documentation in the book of *Animal Genetics Resources in China*: *Pigs* (Wang et al., 2011). Our results suggested that population mobility may be one of the reasons for the establishment of a new breed, which happened passively and accompanied major historical events.

Body weight is a polygenic trait controlled by many genes with minor additive effects. To identify genes associated with body weight in animals generally requires an advanced intercross population and a large number of descendants. Since the body weight of DHB is significantly higher than other breeds from the place to live, with a clear history of genetic introgression of DHB, we therefore sought out and narrowed down candidate genes controlling body weight from the identified introgressed regions. It constitutes a cost‐efficient approach for gene mapping compared with traditional GWAS. A similar approach was previously employed by Bosse et al. (2015) to locate phenotypically important genes introgressed in European commercial pig breeds.

In most cases, the initial admixture proportion would not be high in the recipient population. Introgressed genetic materials that failed to contribute positively to the fitness of the recipient populations would likely be wiped out by random drifts or negative selection. The introgression signals captured by our haplotype similarity method are thus more likely to represent adaptive introgressions. This is supported by the overrepresentation of overlaps of introgression blocks from multiple large‐sized breeds, as well as the observation that introgression regions in DHB are highly divergent to small‐sized breeds. The analyses of the introgression route showed that it was more likely that the beneficial genes were transmitted originally from the north since the introgressed genes from the middle‐upper area of the introgression route were shown under stronger selections.

By analyzing additional resequencing data, we pinpointed candidate haplotypes and mutations from biologically relevant genes that may have an effect on body weight. We also found that candidate genes were enriched in blood circulation (GO: 0008015) and cardiac muscle contraction (GO: 0060048), suggesting that besides muscle development, body weight may also be related to improved blood circulation. Besides body weight, spine shape also differs between large‐sized and small‐sized breeds, as illustrated in Figure 1. The spine of large‐sized breeds and DHB tends to be straight, while the spine of small‐sized breeds appears to be curved. The gene *ACP2*, which controls the thoracic spine shape in mice (Saftig et al., 1997), was found to be introgressed from the large‐sized breeds to DHB (Table S3). However, it is yet unclear if the spine shape is associated with body weight or if it is an independent trait under selection in the DHB.

In conclusion, we reported human migration waves reflected in the hybrid genome composition of DHB pig. We also reported candidate genes and mutations that potentially affected body weight in DHB by introgression and functional analysis. Together we provide compelling evidence of the impact of human migration and selection on the formation of this unique Chinese pig breed.

## CONFLICT OF INTEREST

The authors have no conflict of interest to declare.

## Supporting information

Figure S1‐S7

Table S1‐S13

## Data Availability

Raw sequencing data that support the findings of this study have been deposited to the NCBI BioProject database under accession PRJNA720268.
