# Supplementary material for: Whole‐genome analysis reveals the hybrid formation of Chinese indigenous DHB pig following human migration
Source: Evol Appl. 2022 Mar 16;15(3):501–14. doi: 10.1111/eva.13366 (PMC8965386; doi:10.1111/eva.13366)
Supplement: Supplementary file 1 — Figure S1‐S7 [file EVA-15-501-s002.docx]

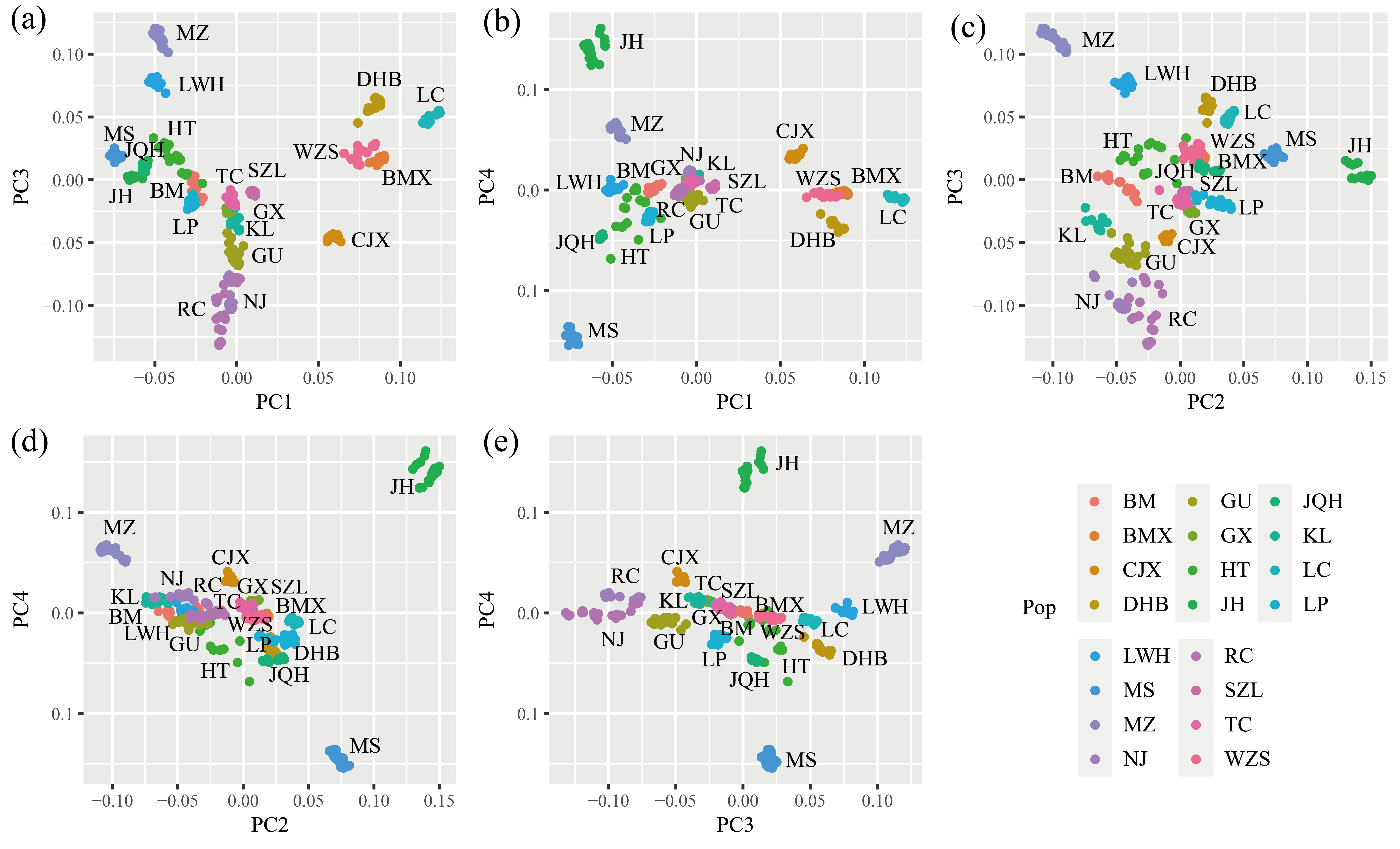


**Figure S1.** PCA results of 20 Chinese pigs ranged from PC1-PC4.


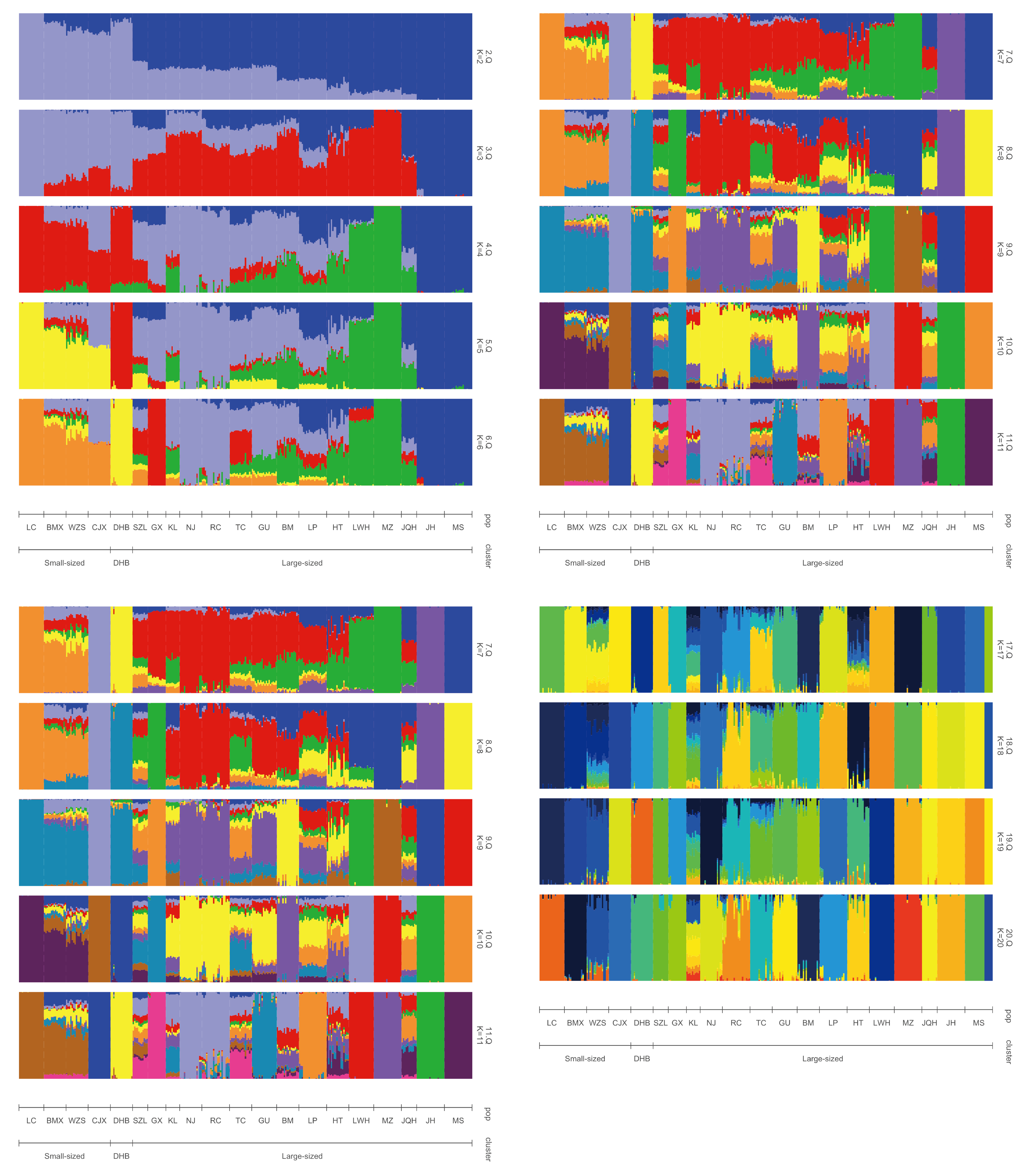


**Figure S2.** Admixture results of 20 Chinese pigs ranged from K=2 to 20.


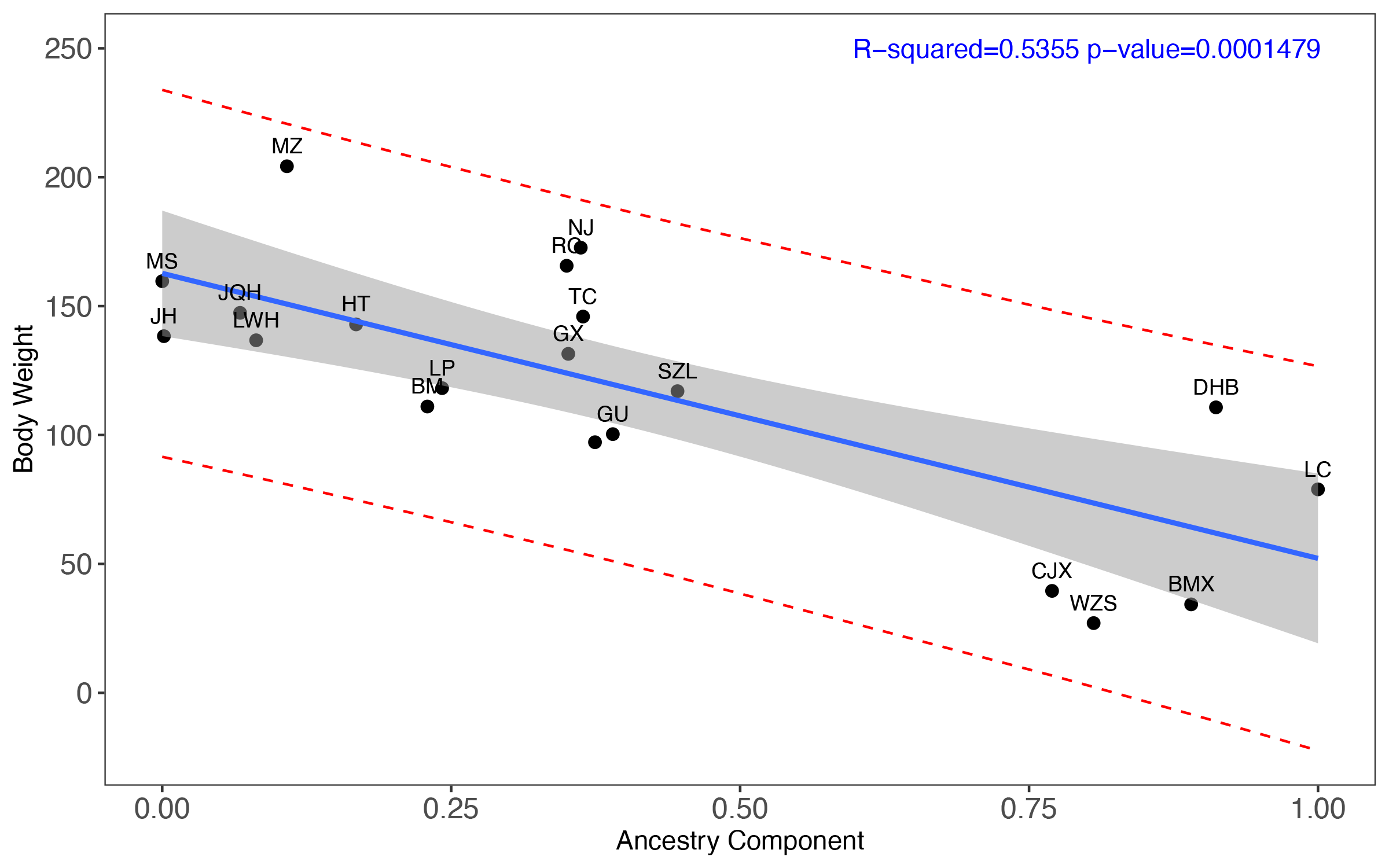


**Figure S3.** Pearson correlation between the ancestral components shared by various breeds with LC and their body weight. The gray area represents the 5%-95% confidence interval. The area between the two red dotted lines represents the 5%-95% prediction interval.


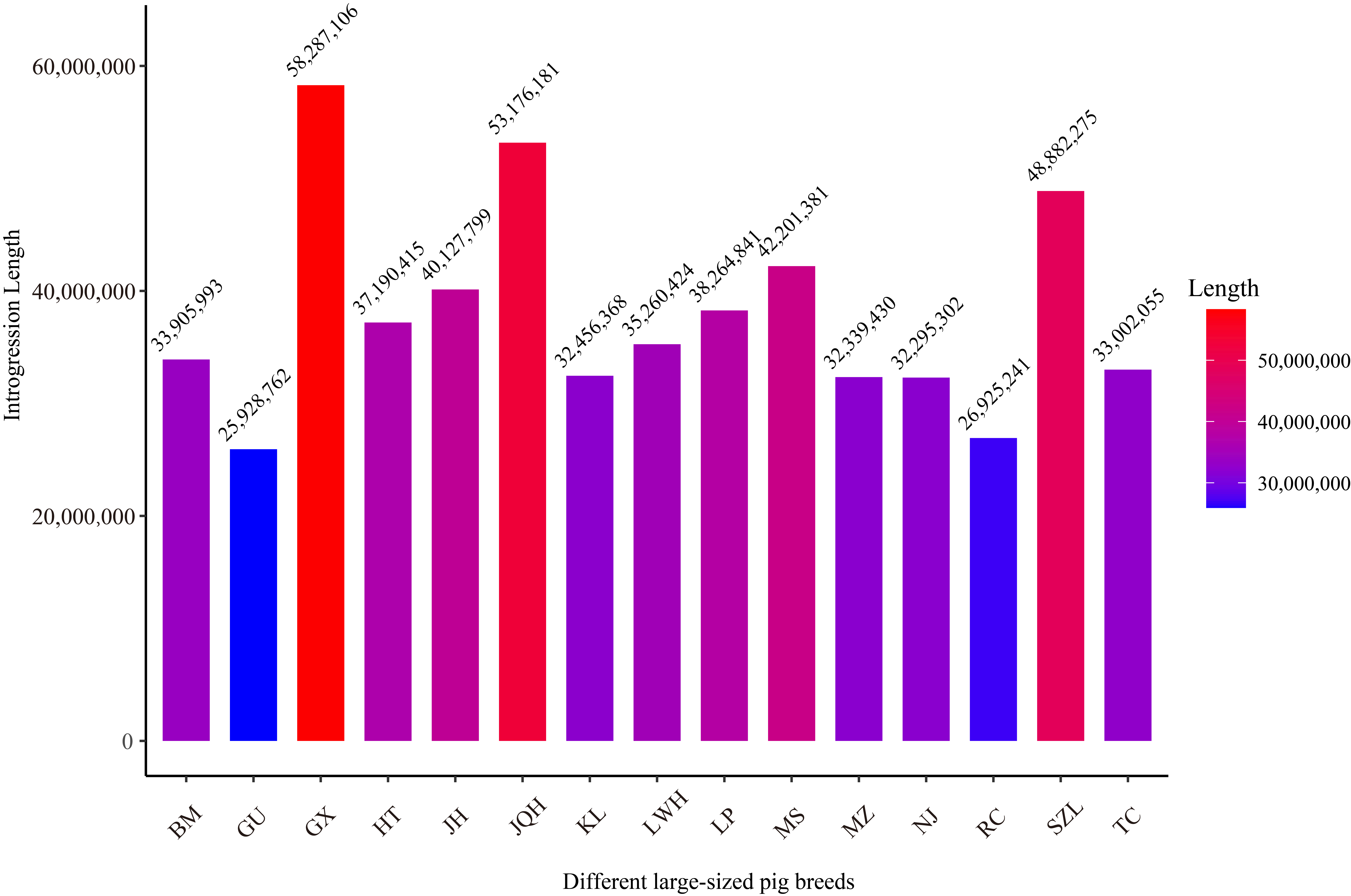


**Figure S4.** The introgression length of different large-sized breeds into DHB.


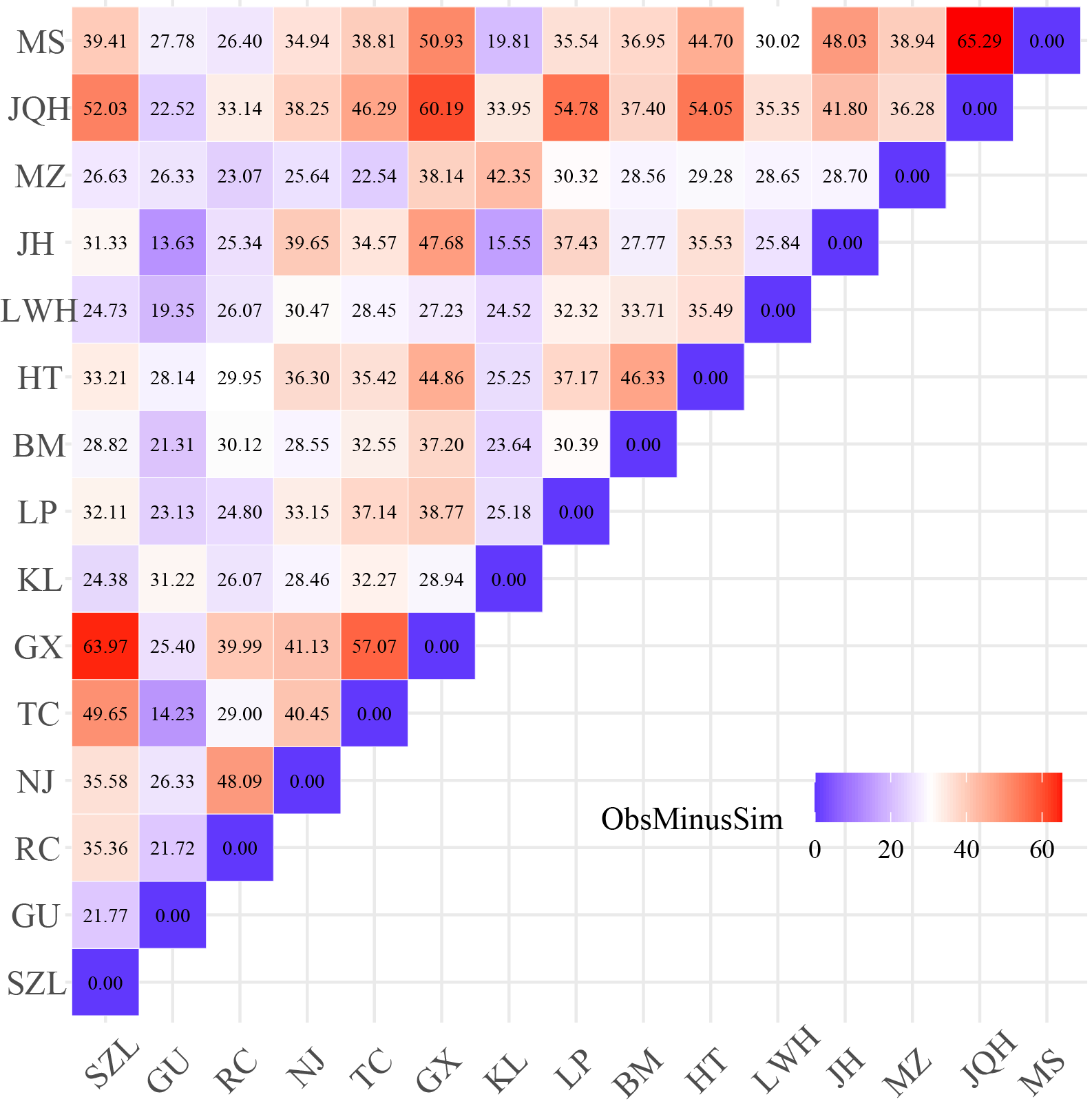


**Figure S5.** The overrepresentation of introgression blocks between two large-sized breeds. The difference between the observed value of the introgression overlapping blocks between two large-sized breeds and the average value of the random sampling distribution is used to measure the degree of deviation of the introgression overlapping blocks from the random sampling distribution in pairs of two large-sized breeds. There are extensive gene flow between local pigs in China and we can observe that most of the introgression blocks between large-sized breeds exceed expectations.


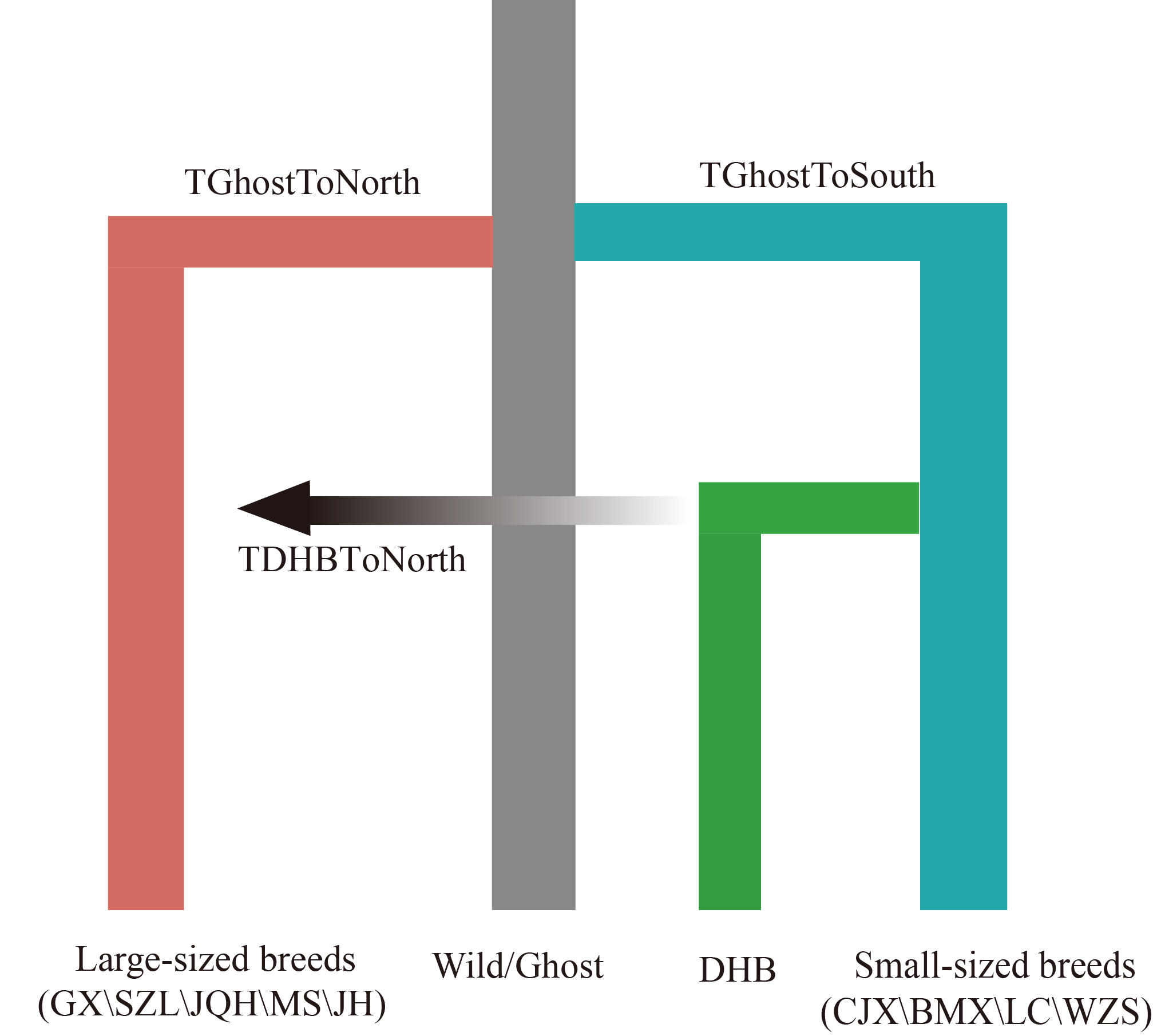


**Figure S6.** Demographic model of DHB breed formation. In this model, DHB is derived from small-sized breeds from the south. And the introgression direction was DHB to large-sized breeds.


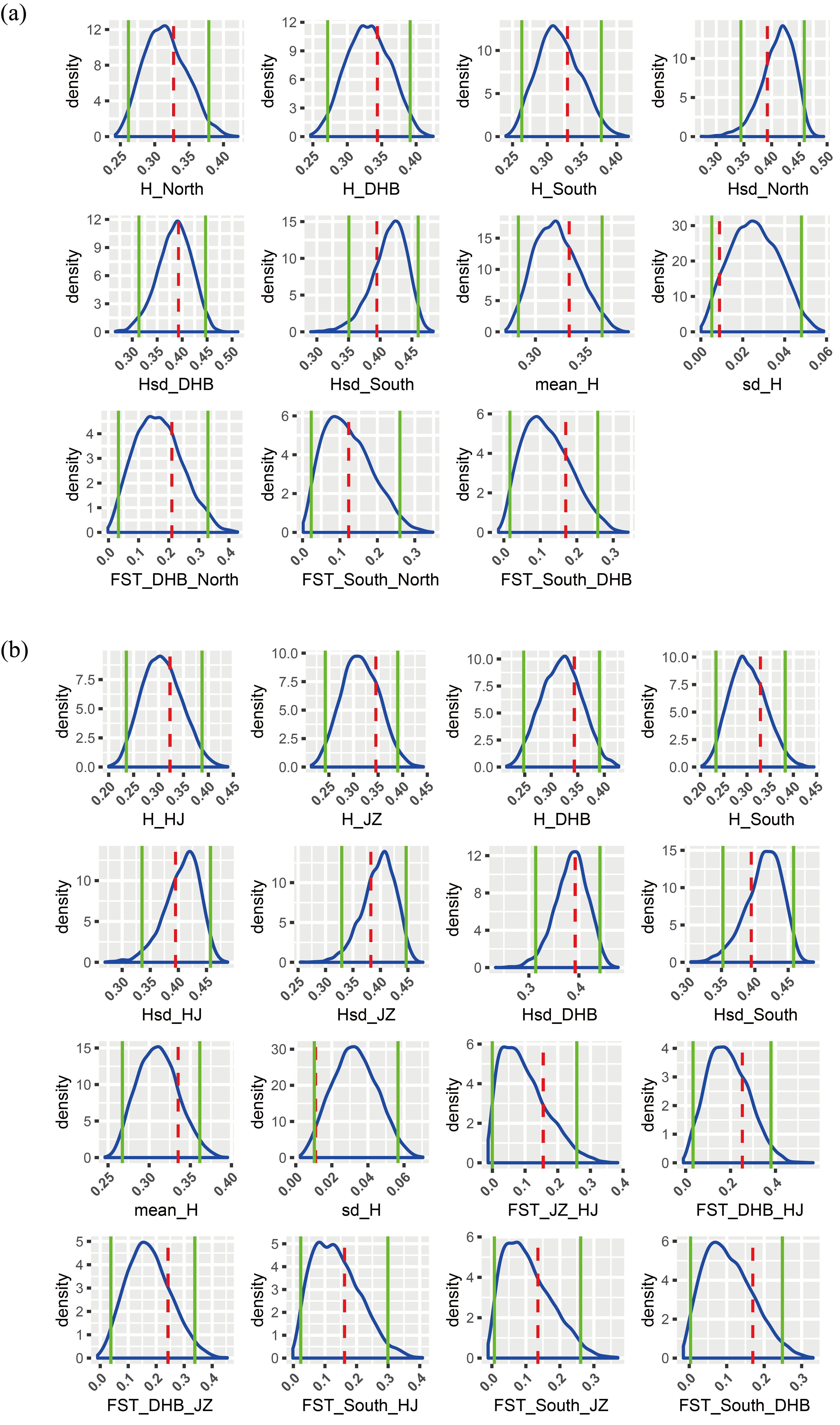


**Figure S7.**Observed and simulated values of ABC models. The red dashes line represents the observed value, and the green solid line represents the simulated value, range of 2.5-97.5%. (a) Statistics for Model in Figure 6a. (b) Statistics for Model in Figure 6b.
